# Reconstruction of a Giant Main Pulmonary Artery Aneurysm Using a Porcine Root in an Adult Patient

**DOI:** 10.7759/cureus.41752

**Published:** 2023-07-12

**Authors:** Besart Cuko, Othmane Haddani, Massimo Baudo, Olivier Busuttil, Louis Labrousse

**Affiliations:** 1 Department of Cardiology and Cardio-Vascular Surgery, Hopital Cardiologique de Haut-Leveque, Pessac, FRA; 2 Department of Cardiac Surgery, ASST Spedali Civili di Brescia, University of Brescia, Brescia, ITA

**Keywords:** case report, surgical case reports, pulmonary artery pseudo aneurysm, medtronic freestyle bioprosthesis, porcine root, pulmonary root replacement, pulmonary artery aneurysm

## Abstract

Pulmonary artery aneurysm (PAA) is a rare disorder with a difficult diagnosis and debated management in literature due to the limited number of cases. Even if the definitive treatment of PAA is surgery, consistent guidelines still need to be developed to help surgeons determine when intervention is appropriate. We report a case of a 77-year-old female diagnosed with central PAA measuring 61 mm at contrast-enhanced computerized tomography scan which was treated surgically. The patient underwent a successful elective complete pulmonary root replacement with a Medtronic Freestyle (Medtronic Inc, Minneapolis, MN) porcine root. Postprocedural recovery and follow-up at 12 months were uneventful.

## Introduction

Aneurysms involving the main pulmonary artery and/or its branches are rare conditions with limited clinical occurrence. Also, its pathogenesis is poorly understood due to non-specific symptoms. Frequently, there is a complex interplay of multiple factors that lead to pulmonary artery aneurysm (PAA) formation. Management of PAAs is variable and generally depends on the underlying etiology, size of the aneurism, and patient’s comorbidities. Surgery is recommended when the patient is symptomatic and the diameter of the pulmonary artery is greater than 5 cm, as the risk of rupture is associated with a nearly 50% mortality rate [1]. We report a case of a giant PAA successfully treated surgically with reconstruction using a Medtronic Freestyle (Medtronic Inc, Minneapolis, MN) porcine root in an adult patient.

## Case presentation

**Figure 1:**
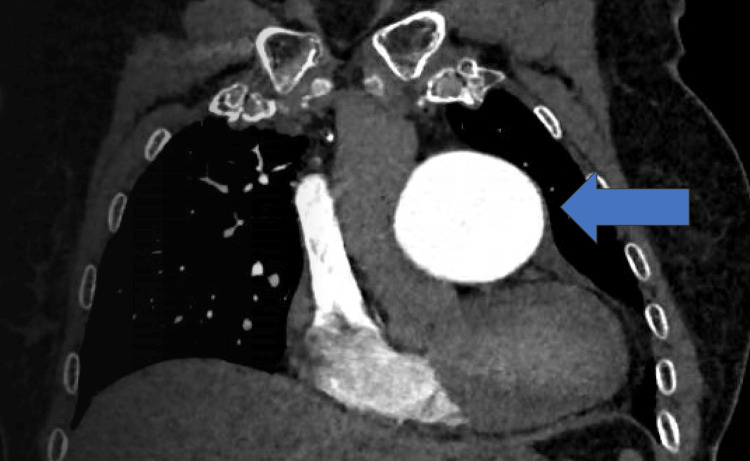
Coronal plane of pre-operative CT scan showing a giant PAA PAA: Pulmonary artery aneurysm

**Figure 2:**
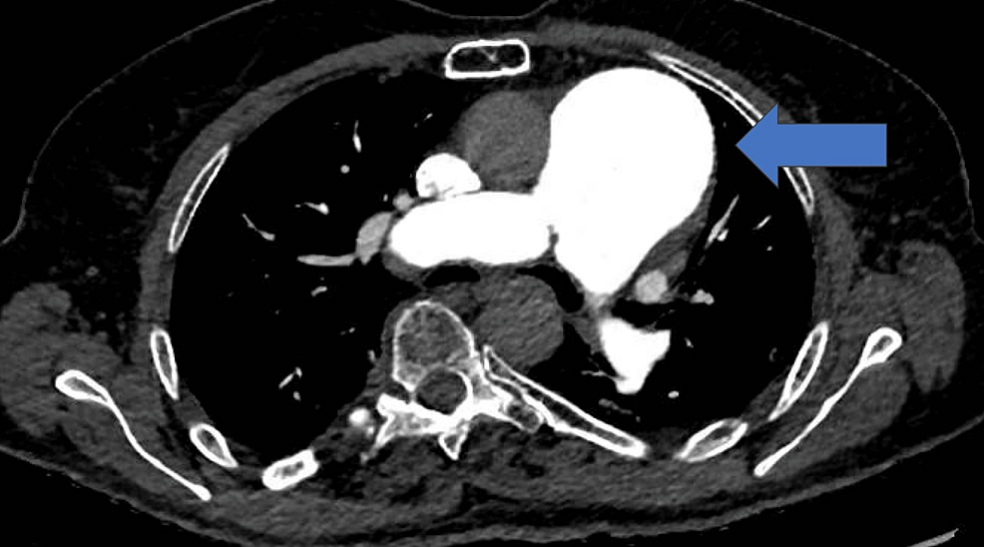
Axial plane of pre-operative CT scan showing a giant PAA PAA: Pulmonary artery aneurysm

**Figure 3:**
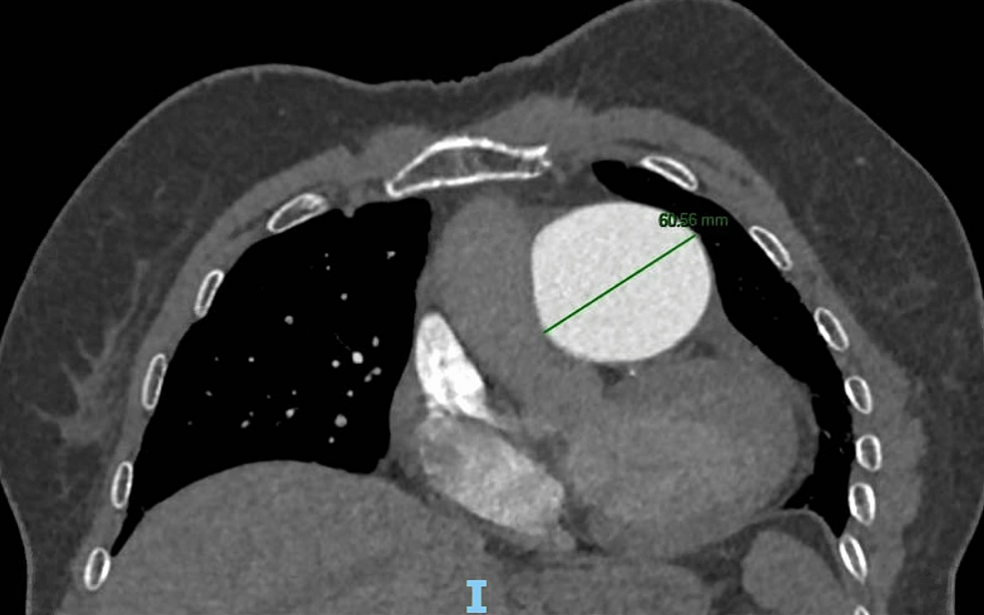
Maximum diameter of PAA on axial plane of pre-operative CT scan PAA: Pulmonary artery aneurysm

A 77-year-old female was admitted to our institution after the diagnosis of a central PAA with a maximum diameter measuring 61 mm at contrast-enhanced computerized tomography (ce-CT) scan performed for worsening dyspnea (Figure 1-3; the axial and coronal plane of pre-operative ce-CT scan). 

Her medical history included arterial hypertension treated with irbesartan and ivabradine, left breast cancer treated with surgery and radiotherapy, glaucoma, and scoliosis. Transthoracic (TTE) and transesophageal (TEE) echocardiography showed normal biventricular function in the presence of mild-to-moderate pulmonary valve regurgitation associated with PAA. Right heart catheterization excluded pulmonary arterial hypertension. Autoimmune, inflammatory, and infective investigations were totally negative, and no signs of connective tissue diseases were present. A complementary positron emission tomography/computed tomography (PET/CT) was performed that was within normal limits, without any difference in metabolic activity between the aorta and the pulmonary artery. After the Heart Team assessment, we decided to proceed for a surgical treatment.

**Figure 4:**
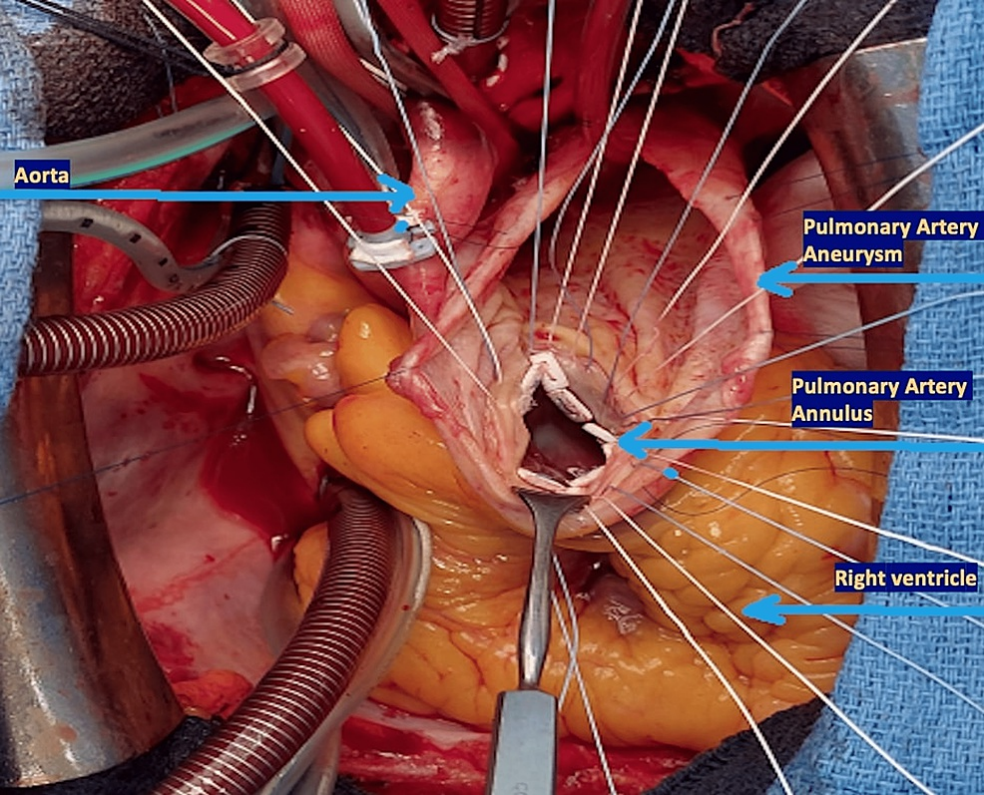
PAA and pulmonary valve leaflets resected PAA: Pulmonary artery aneurysm

**Figure 5:**
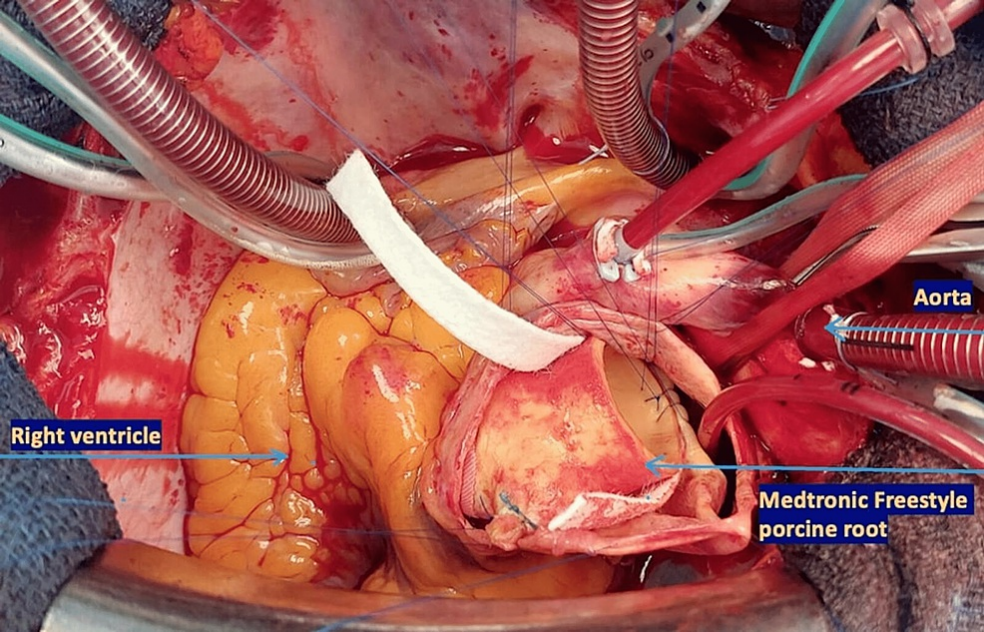
Medtronic Freestyle Nr. 29 (Medtronic Inc) porcine root

Under general anesthesia, a full longitudinal sternotomy was performed with evidence of a giant and translucent central PAA. The cardiopulmonary bypass (CPB) was established by aortic cannulation for the arterial line and bicaval cannulation for the venous line. After infusion of the cardioplegia solution, an incision of the PAA was performed and the pulmonary valve was inspected. The valve was tri-leaflet with leaflets thickening and significantly reduced coaptation causing pulmonary valve regurgitation. A radical replacement was decided and a full pulmonary root replacement using a Medtronic Freestyle Nr. 29 (Medtronic Inc) bioprosthesis was performed (Figure 4 and Figure 5).

**Figure 6:**
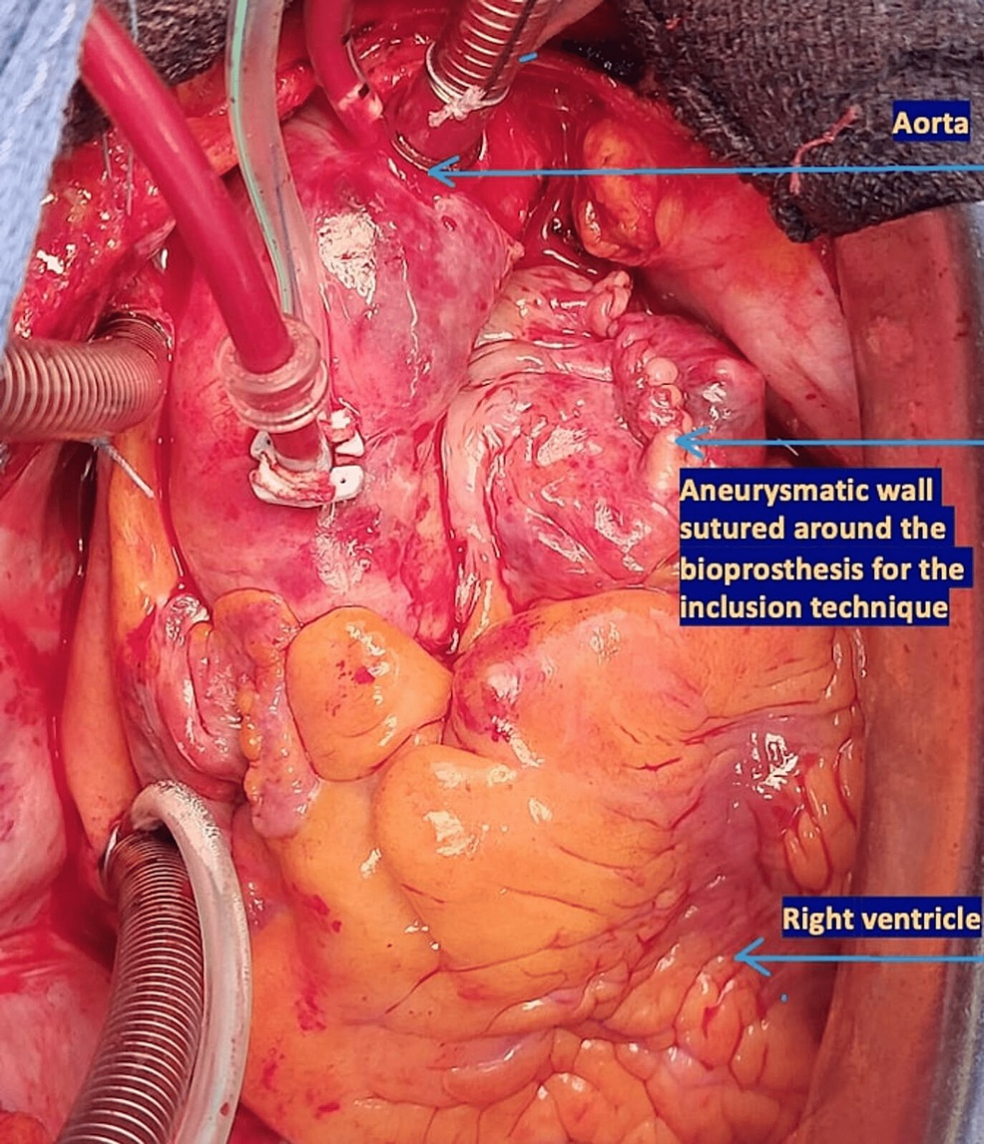
Redundant aneurysmatic wall sutured around the bioprosthesis at the end of the procedure

At the end of the procedure, the redundant aneurysmatic wall was sutured around the porcine root as shown in Figure 6.

The intraoperative TEE was satisfactory with good blood flow in both pulmonary arteries. Postprocedural recovery was uneventful with a good hemodynamic response and the patient was discharged home on postoperative day 7. Follow-up at 12 months, including a CT scan, showed excellent surgical results and total recovery of the New York Heart Association (NYHA) functional class. The patient’s informed consent for the procedure and for data collection were obtained.

## Discussion

PAAs are usually asymptomatic and diagnosed as incidental findings due to the increased use of imaging diagnostic tools, especially CT chest. PAAs are classified as true or pseudoaneurysms. A true aneurysm involves all three layers of the vascular wall, whereas a pseudoaneurysm involves only the external layers, with an intrinsically higher risk of rupture than a true aneurysm. Based on the anatomical location, PAAs can be central, involving the main pulmonary artery, or peripheral, when segmental or subsegmental arteries are involved. The average adult pulmonary artery diameter is 25 mm ± 3 mm with the upper limit of normality being 29 mm in males and 27 mm in females [2]. PAA is defined as a 1.5 times minimum dilation of the normal diameter of the pulmonary artery, involving all three layers of the vessel wall [3]. PAA clinical manifestations and pathogenesis are poorly understood due to non-specific symptoms ranging from massive hemoptysis or sudden death to the most common presenting symptoms like dyspnea, pleuritic chest pain, and cough [4]. Similarly, our patient also presented with worsening dyspnea. TTE is an important tool for the diagnosis of central PAA but has a limited role in evaluating peripheral PAAs. Ce-CT scan is the gold standard imaging tool to evaluate the morphology and the location of PAAs and to differentiate it from other vascular pathologies [5]. PAAs may be associated with pulmonary valve pathologies, as shown by Reisenauer et al. that the most common pulmonary valve pathology associated with PAA is pulmonary valve regurgitation [6]. There are no clear guidelines for the best treatment of PAAs, but surgery is commonly considered in patients with pulmonary trunk aneurysms greater than 5 cm, especially in symptomatic patients. Pulmonary artery hypertension or compression of adjacent structures are other suggested surgical indications [7]. Turnow et al. described a case report of effective conservative management of a large idiopathic PAA with a 15% decrease in size at a 6-month follow-up in an asymptomatic and hemodynamically stable patient [8]. However, when conservative management is chosen, aneurysm rupture is possible and consequences may be lethal. So, we believe that once the diagnosis of PAA is made, surgical treatment should be pursued when feasible. Our patient was a 77-year-old female with central PAA associated with pulmonary valve regurgitation and worsening dyspnea. We considered the risk of intervention to be lower than the risk of conservative management, and due to worsening dyspnea, we decided on a radical approach. The surgical approach must consider the location of PAAs and the patient’s comorbidities. Aneurysm resection followed by pulmonary artery reconstruction or pulmonary artery replacement can both be performed. Reisenauer et al. described aneurysmorrhaphy or aneurysmectomy with graft interposition as a surgical option in their single-center experience of 38 patients [6]. Franciosi et al. and Circi et al. described a Yacoub and a David’s procedure to treat a giant PAA in the presence of a normal pulmonary valve, respectively [9,10]. We opted for a porcine root due to its excellent performance and long-term outcomes [11,12]. No matter what kind of operation is adopted, the basic principle is to correct the patient’s pathological changes.

This case shows that a full pulmonary root replacement using a porcine root, as an alternative surgical approach, is feasible in patients with central PAAs and pulmonary regurgitation. Surgical treatment of PAAs should be considered when the surgical risk is considered lower than the risk of conservative management. It is important to underline that multidisciplinary perioperative management and Heart Team assessment are crucial in these patients.

## Conclusions

Radical surgery in PAAs should be considered when the surgical risk is considered lower than the risk of conservative management. Porcine root bioprosthesis can be considered a possible surgical approach of central PAAs among other strategies. Additional studies are needed to develop more reliable approaches and surgical guidelines to manage patients with PAAs.
